# Medullary sponge kidney with IgA nephropathy: a case report and literature review

**DOI:** 10.1186/s12882-024-03596-w

**Published:** 2024-05-03

**Authors:** Chuchu Zeng, Yunjie Jin, Yanzhe Wang, Dingyu Zhu, Zhigang Zhang, Xiaoxia Wang

**Affiliations:** 1 Department of Nephrology, Tongren Hospital, Shanghai Jiao Tong University School of Medicine, 1111 Xianxia Road, Shanghai, 200336 China; 2 Department of Ultrasound, Zhongshan Hospital, Fudan University, Shanghai, China; 3 Department of Pathology, School of Basic Medical Sciences, Fudan University, Shanghai, China

**Keywords:** Medullary spongy kidney, IgA nephropathy, Kidney biopsy, Case report

## Abstract

**Background:**

Medullary sponge kidney (MSK)is rare in association with glomerulonephritis. We report a patient with medullary sponge kidney, and the kidney biopsy revealed a diagnosis of IgA nephropathy.

**Case presentation:**

A 27-year-old female presented with hematuria and proteinuria, and imaging studies indicated the presence of medullary spongy kidney. With appropriate preparation, a kidney biopsy was performed. Considering the patient’s clinical and pathological characteristics, the final diagnosis was determined to be medullary sponge kidney associated by IgA nephropathy. The combination of corticosteroids and angiotensin receptor blockers (ARBs) proved to be significantly effective in reducing proteinuria in the current case. To the best of our knowledge, this is the first reported case that demonstrates the coexistence of MSK and IgA nephropathy.

**Conclusions:**

Administering precise therapy based on renal pathology can potentially enhance outcomes for patients with renal conditions, necessitating the need for clinicians to be vigilant about differential diagnosis in order to reduce the rates of missed diagnoses and misdiagnosis.

## Background

Medullary sponge kidney (MSK) is a congenital anomaly characterized by the dilation of the precalix ducts. It is often linked to renal calculi or calcinosis, defective urinary acidification and concentration, and recurrent urinary tract infections [1, 2]. Nonetheless, the association of MSK with glomerulonephritis is rare [3–6]. In this report, we present a case in which MSK was discovered through renal enhanced CT, and the results of the percutaneous kidney biopsy indicated IgA nephropathy (IgAN). This is the first documented instance of MSK and IgAN coexistence.

## Case presentation

A 27-year-old female with a six-month history of proteinuria close to the nephrotic range (2 +∼3+) and hematuria (2 +∼3+) was admitted to the clinic due to nephritic syndrome. She experienced transient gross hematuria six months ago, and subsequent reviews revealed persistent microscopic hematuria and proteinuria. There were no significant medical conditions in her past medical history. Upon admission, a physical examination showed bilateral mild pretibial edema, while other physical examination findings were normal.

Laboratory studies revealed the following findings: the complete blood count showed a leukocyte count of 6050/mm^3 with 50.2% neutrophils. The hematocrit was 35% with a hemoglobin level of 12.3 g/dL. Erythrocyte sedimentation rate (ESR) in the first hour was 42 mm. The C-reactive protein (CRP) level was 0.13 mg/L. Serum chemistry indicated a blood urea nitrogen level of 79.6 mg/dL, a serum creatinine level of 0.80 mg/dL, a low total serum protein level of 6.1 g/dL, an albumin level of 3.2 g/dL, and a total cholesterol level of 122 mg/dL. Furthermore, immunological tests showed a reduced serum IgG level of 774 mg/dL (normal range:860–1740 mg/dL), while the serum IgA, IgM, C3, and C4 levels were within normal range (239, 149, 97, and 23 mg/dL, respectively). Other serological tests yielded negative results for antinuclear antibodies, anti-double-stranded DNA antibodies, anti-neutrophil cytoplasmic antibody, anti-GBM antibodies, anti-Smith antibodies, and anti-phospholipase A2 antibodies. Serological markers for hepatitis B and C viruses were negative, as were serum tumor markers including carbohydrate antigen 19 − 9, alpha-fetoprotein, and carcinoembryonic antigen.

**Fig. 1:**
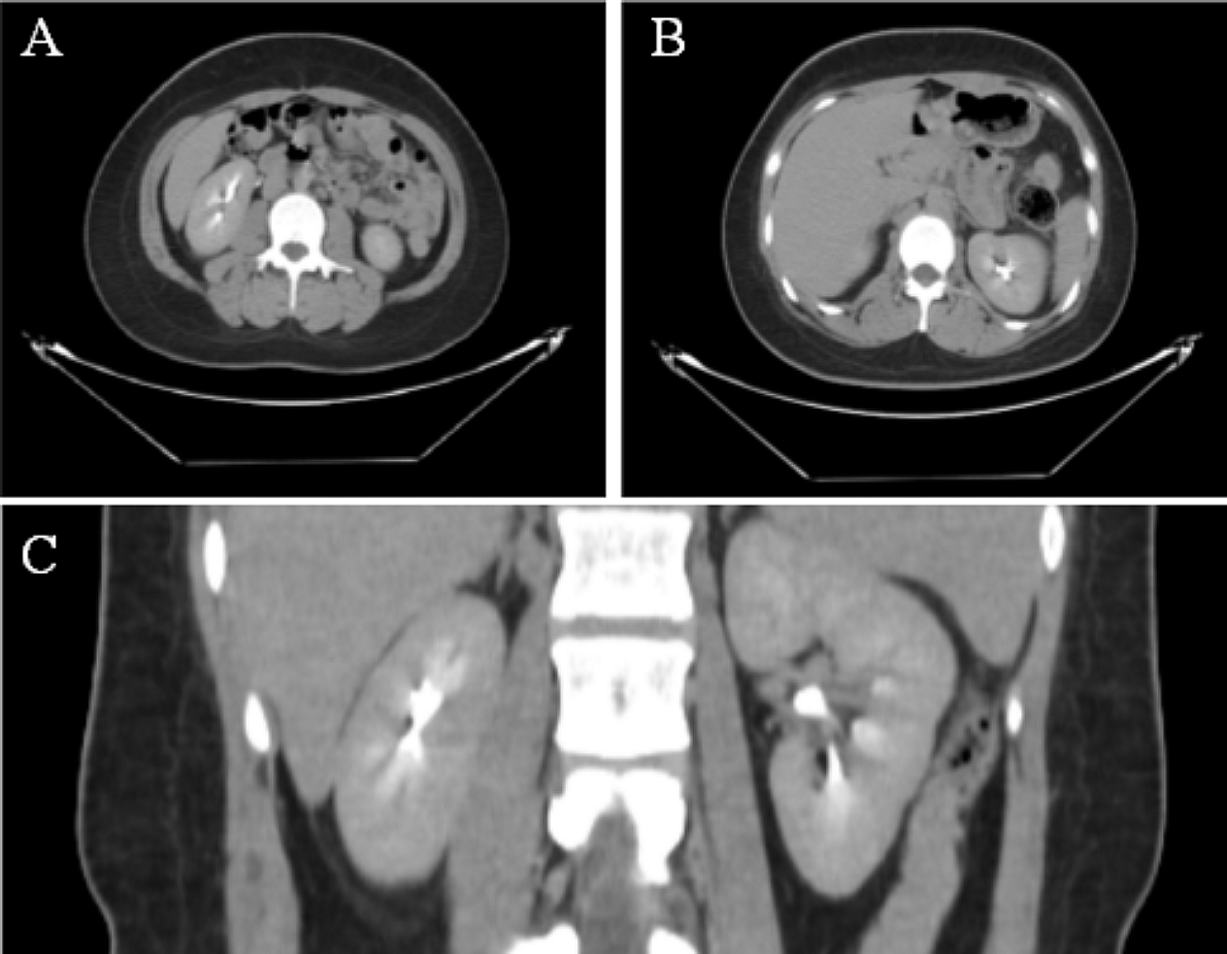
Enhancement CT of urinary system. Increased diffuse density in the medullary area of both kidneys with small stones. **(A, B) **Cross-section. **(C)** Coronal plane

Urine analysis revealed hematuria (3+), proteinuria (2+), protein levels of 3230 mg/24 h, albuminuria levels of 2489 mg/24h, urine density of 1.013, and urine pH of 7. Abdominal ultrasound showed multiple small bilateral renal calculi without hydronephrosis and pyramidal echogenic foci consistent with MSK in both kidneys. Enhanced computed tomography (CT) scan showed increased diffuse density in the medullary area of both kidneys with small stones, leading to the diagnosis of MSK (Fig. 1).

**Fig. 2:**
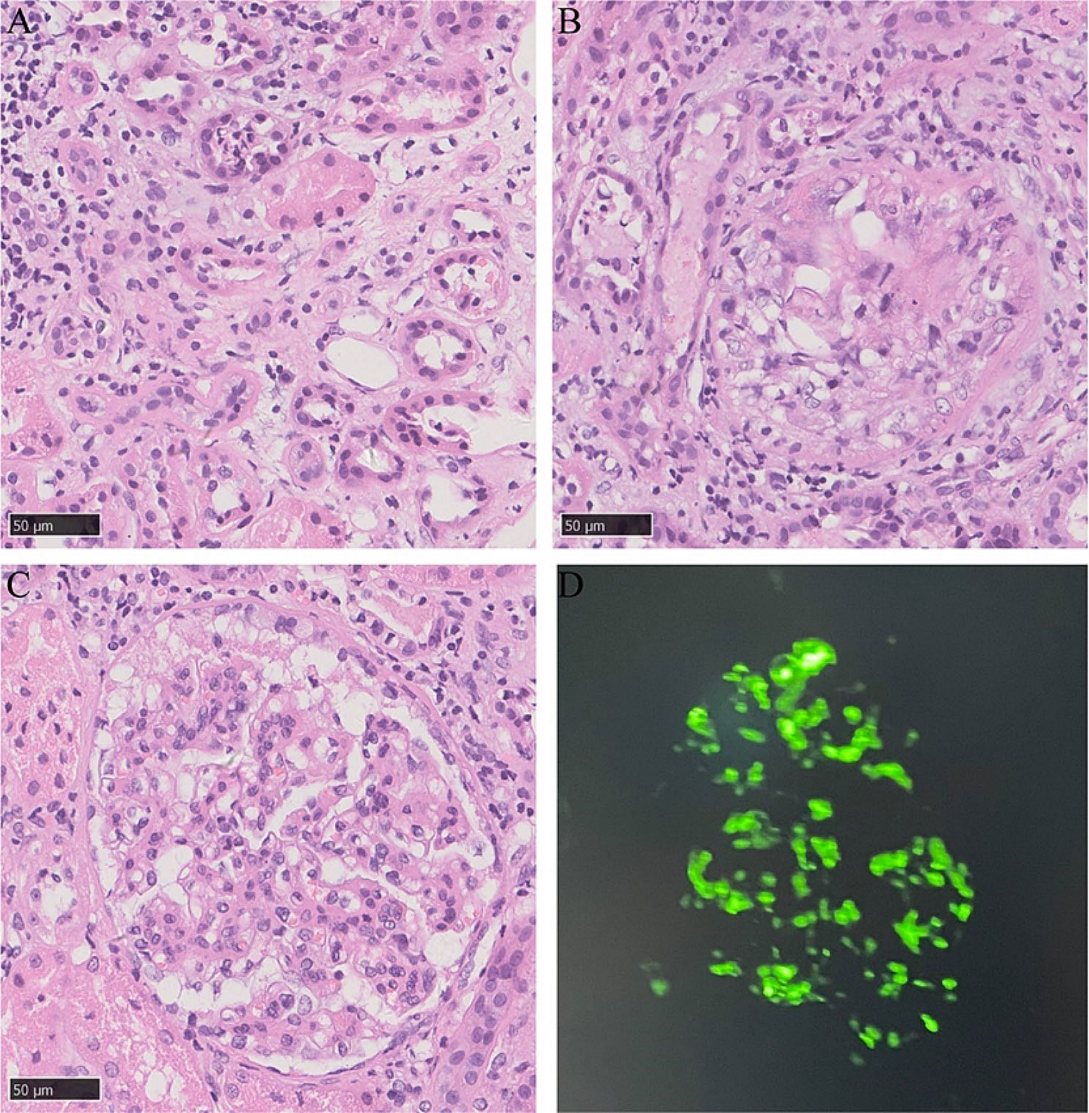
The light microscope results **(A, B, C)** and Immunofluorescent analysis results **(D)** of the patient. **(A)** Inflammatory cell infiltration in the interstitium(×400). **(B)** Cellular crescent formation was present in 1 glomeruli(×400). **(C)** Mesangial cell proliferation and mesangial matrix expansion (×400). **(D)** Diffuse IgA (2+) staining across several mesangial areas (×400)

**Fig. 3:**
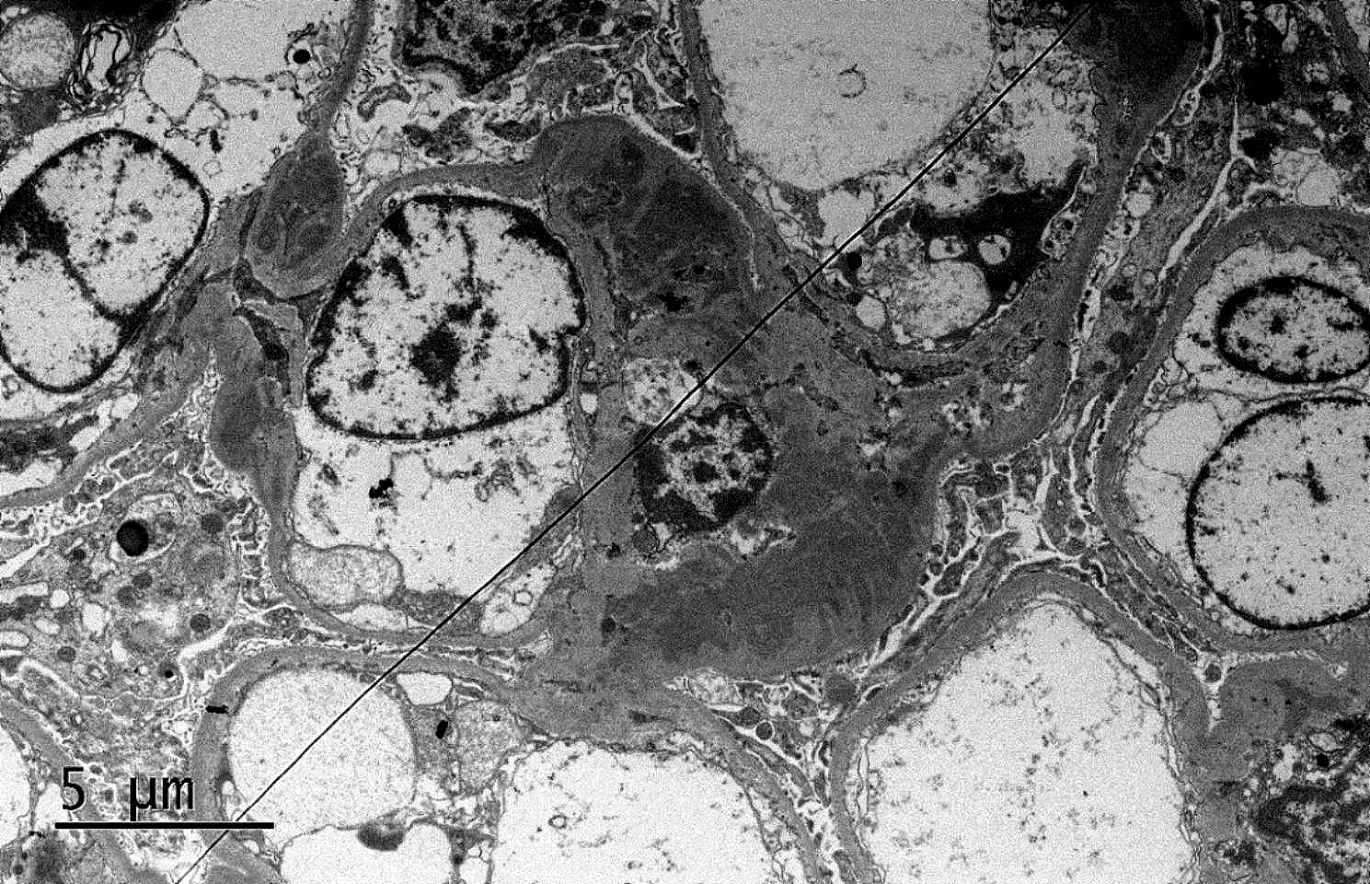
The electron microscope results of the patient. Electron-dense material in the mesangial region of the glomerulus and partially foot process effacement of the podocyte(×5,000)

A kidney needle biopsy was scheduled, given the proteinuria (3.23 g). Light microscopy revealed sclerosing lesions in 1 out of 10 glomeruli, with mild to moderate mesangial cell proliferation and moderate mesangial matrix expansion observed in some glomerular segments. Cellular crescent formation was present in 3 glomeruli, and fibrous crescent formation in 1 glomerulus. Renal tubules displayed localized atrophy, inflammatory cell infiltration in the interstitium, while the renal interstitial vessels appeared unremarkable. Immunofluorescent analysis showed diffuse IgA (2+) and C3 (+) staining across several mesangial areas (Fig. 2). The pathological diagnosis was focal segmental hyperplasia IgAN, according to the Oxford classification (M1E0S1T0C1). Electron microscopy examination showed there were more deposits of electron-dense material in the mesangial region of the glomerulus, the matrix in the mesangial region was mild to moderate increase increased, and partially foot process effacement of the podocyte (Fig. 3).

We administered a combination therapy of oral prednisolone (40 mg qd, the subsequent dose was tapered) and losartan potassium Table (100 mg qd) to the patient. Following five months of treatment, the patient exhibited a partial remission of proteinuria and experienced relief from hematuria. The levels of serum albumin, serum creatinine, urine protein and urine erythrocyte of this patient on different dates are show in Table 1. The patient is currently undergoing treatment.

**Table 1:** The levels of serum albumin, serum creatinine, urine protein and urine erythrocyte on different dates

	Jun.27.23	Oct.24.23	Nov.14.23	Dec.23.23
Albumin (g/dL)	3.2	3.7	No available	No available
Creatinine (mg/dL)	0.80	0.86	No available	No available
Urine protein (g/24 h)	3.23	2.37	0.67	0.88
Urine erythrocyte (/uL)	530	117	13	59

## Discussion and conclusion

We present the case of a young female patient who exhibited hematuria and proteinuria as the primary clinical presentations. Imaging examinations suggested the presence of medullary sponge kidney (MSK). Kidney biopsy pathology confirmed the diagnosis of IgAN with focal segmental hyperplasia (segmental proliferation of mesangial cells in glomeruli). Based on the clinical and pathological findings, the final diagnosis was MSK associated with IgAN.

MSK is a congenital renal dysplasia characterized by the sponge-like dilation of the papillary and collecting ducts within the renal pyramids. Its estimated incidence in the general population ranges from 1/20,000 to 1/5,000 [7]. The first description of MSK dates back to 1939 by Lanarduzzi, with subsequent detailed documentation by Cacchi and Ricci in 1948 [8]. The exact etiology and pathogenesis of MSK are not yet fully understood. However, recent scientific advancements in genetics have shed light on the study of MSK [9, 10].

The clinical presentations associated with MSK include renal calculi, medullary cystic dilatation, concentration defects, renal tubular acidosis, and recurrent urinary tract infections. Several developmental disorders such as hemihypertrophy, Beckwith-Wiedemann syndrome, autosomal dominant polycystic kidney disease, horseshoe kidney, and renal arterial fibromuscular dysplasia have been reported as renal associations of MSK [8, 11]. However, the coexistence of MSK with glomerulonephritis is rare (Table 2). Levitt (1974) reported a 48-year-old woman in whom progressive renal failure was noted terminated in bilateral nephrectomy and living donor transplantation that was later diagnosed with focal sclerosing glomerulopathy and MSK [3]. In 2003, Prencipe reported a case of MSK complicated by mesangial glomerulonephritis [4]. Akoglu and Fatih also described patients with MSK presenting with nephrotic-range proteinuria, which was later diagnosed as renal AA amyloidosis [5, 6]. To the best of our knowledge, the patient we present is the first documented case demonstrating the coexistence of MSK and IgAN.

**Table 2:** Features of the patients with MSK and glomerulonephritis reported in the literature and the present case

Age(y), gender and references	Published year	Types of glomerulonephritis	Methods of harvesting kidney tissue	Clinical features	Diagnosis of MSK
48, Female^3^	1974	Focal sclerosing glomerulopathy	Bilateral nephrectomy and living donor transplantation.	Progressive renal failure	A complete radiologic evidence
No available^4^	2003	Mesangial glomerulonephritis	Renal needle biopsy	No available	RX urography and renal TC
43, Female^5^	2010	renal AA amyloidosis	Renal needle biopsy	Hematuria and nephrotic-range proteinuria	RX urography and renal TC
28, Male^6^	2022	renal AA amyloidosis	Renal needle biopsy	proteinuria	Abdominal ultrasound

Most patients with MSK have a favorable prognosis and rarely experience significant proteinuria or renal impairment. The patient in question exhibited persistent microscopic hematuria alongside proteinuria close to the nephrotic range. Therefore, it was suggested that the patient not only exhibited pathological changes associated with medullary sponge kidney but also potentially had complications involving glomerular lesions. There have been differing viewpoints among experts regarding the issue of performing percutaneous kidney biopsy on patients with MSK, both in domestic and foreign literature. After thoroughly evaluating the benefits and risks and making adequate preparations, we successfully performed ultrasound-guided percutaneous kidney biopsy to determine the underlying pathology. This approach was crucial for achieving an accurate diagnosis and implementing timely interventions to prevent further decline in renal function.

Therefore, administering precise therapy based on renal pathology can potentially enhance outcomes for patients with renal conditions, necessitating the need for clinicians to be vigilant about differential diagnosis in order to reduce the rates of missed diagnoses and misdiagnosis.

## Data Availability

The datasets used and/or analyzed during the case report are available from the corresponding author on reasonable request.
